# Genotyping of *Enterocytozoon bieneusi* and Subtyping of *Blastocystis* in Cancer Patients: Relationship to Diarrhea and Assessment of Zoonotic Transmission

**DOI:** 10.3389/fmicb.2017.01835

**Published:** 2017-09-21

**Authors:** Weizhe Zhang, Guangxu Ren, Wei Zhao, Ziyin Yang, Yujuan Shen, Yihua Sun, Aiqin Liu, Jianping Cao

**Affiliations:** ^1^Department of Parasitology, Harbin Medical University Harbin, China; ^2^National Institute of Parasitic Diseases, Chinese Center for Disease Control and Prevention, Key Laboratory of Parasite and Vector Biology, Ministry of Health, WHO Collaborating Centre for Malaria, Schistosomiasis and Filariasis Shanghai, China; ^3^Department of Clinical Laboratory, Third Affiliated Hospital of Harbin Medical University Harbin, China

**Keywords:** *Enterocytozoon bieneusi*, *Blastocystis*, cancer patients, genotyping, subtyping

## Abstract

*Enterocytozoon bieneusi* (*E. bieneusi*) and *Blastocystis* are common pathogens responsible for diarrhea in humans, especially in immunocompromised individuals. The number of cancer patients has been increasing and diarrhea is a common clinical symptom in the treatment of cancers. To understand the prevalences and genotypes/subtypes of *E. bieneusi* and *Blastocystis* in cancer patients in China, to track the infection sources, and to explore the relationships between *E. bieneusi* and *Blastocystis* infections and diarrhea, 381 fecal specimens were collected from cancer patients. Each of them was analyzed for the presence of *E. bieneusi* and *Blastocystis* by PCR amplifying and sequencing the ITS region of the rRNA gene and the barcode region of the SSU rRNA gene, respectively. 1.3 and 7.1% of cancer patients were positive for *E. bieneusi* and *Blastocystis*, respectively. No statistical differences were observed in the infection rates between the groups by age, gender, and residence. *E. bieneusi* and *Blastocystis* were both significantly more common in cancer patients with diarrhea, and significant relationship of *Blastocystis* to diarrhea was found in chemotherapy group. Two *E. bieneusi* genotypes (D and a novel one named as HLJ-CP1) and two *Blastocystis* subtypes (ST1 and ST3) were identified with three novel ST1 sequences. This is the first report of occurrence and molecular characterizations of *E. bieneusi* and *Blastocystis* in cancer patients in China. *E. bieneusi* genotype D and *Blastocystis* ST1 and ST3 have been identified in humans and animals while one novel *E. bieneusi* genotype falling into zoonotic group 1, implying a potential of zoonotic transmission.

## Introduction

Microsporidia, known as emerging and opportunistic pathogens, are obligate intracellular parasitic eukaryotes that infect different invertebrate and vertebrate hosts (human, domestic, and wild animals). To date, 17 species in nine genera have been identified as capable agents of producing pathology in humans among ~1,300 species belonging to at least 160 genera (Deng et al., 2016). *Enterocytozoon bieneusi* (*E. bieneusi*) is the most common species in humans, which can be identified in more than 90% of human microsporidiosis cases (Zhao et al., 2014a). *E. bieneusi* causes a self-limiting diarrhea, sometimes appears asymptomatic in immunocompetent persons (Didier, 2005; Matos et al., 2012). However, it may cause persistent diarrhea, malabsorption and weight loss in immunocompromised individuals, even life-threatening diarrhea in AIDS patients (Waywa et al., 2001; Matos et al., 2012). Currently, based on sequence analysis of the internal transcribed spacer (ITS) region of the rRNA gene of *E. bieneusi* isolates, more than 240 ITS genotypes have been identified (Santín and Fayer, 2011; Karim et al., 2014). At least 70 genotypes have been found in humans, with 33 being zoonotic genotypes (Yang et al., 2016).

*Blastocystis* is a common intestinal parasite infecting humans and many different animal species. Recent studies “*in vitro*” and “*in vivo*” have shed new light on the pathogenic power of this parasite. *Blastocystis* infection is associated with a variety of gastrointestinal disorders, irritable bowel syndrome (IBS), and cutaneous lesions (Wawrzyniak et al., 2013). Moreover, the patients with *Blastocystis* as the only detected possible pathogen were observed to relieve from gastrointestinal symptoms after successful treatment of *Blastocystis* (Idris et al., 2010; Vogelberg et al., 2010; Dinleyici et al., 2011). This parasite is frequently found in immunocompromised individuals, showing its characteristic of opportunistic pathogenesis (Wawrzyniak et al., 2013). Molecular data reveal extensive genetic diversity within *Blastocystis* genus in the small subunit (SSU) rRNA gene. To date, 17 subtypes (STs) have been reported based on sequence analysis of the SSU rRNA gene, and nine of them have been identified in humans, with eight being zoonotic (Stensvold, 2013).

In recent years, the number of cancer patients has been increasing. In 2013, there were 14.9 million incident cancer cases and 8.2 million cancer deaths worldwide (Global Burden of Disease Cancer Collaboration et al., 2015). In 2015, it was estimated that ~4.3 million new cancer cases and 2.8 million cancer deaths would occurred in China (Chen et al., 2016). Currently, chemotherapy as one of the most effective means to treat cancer is reported to possibly down-regulate the immunity of the patients and increases the risk of parasitic infections (Solomayer et al., 2003). In addition, *Encephalitozoon* microsporidia infection could induce cancer by impairing the transcriptional function of p53 which plays a pivotal role in the control of cell cycle arrest and apoptosis (del Aguila et al., 2006). One study has shown that *Blastocystis* might facilitate tumor development based on the fact that increased cell proliferation in different cancer cell lines after incubation with soluble *Blastocystis* antigens (Kumarasamy et al., 2014).

Currently, there have been a few reports of microsporidia and *Blastocystis* infections in cancer patients in Iran, Mexico, Malaysia, France, and Turkey, with genotypes D and E of *E. bieneusi* and ST1-ST7 of *Blastocystis* being identified (Lono et al., 2008; Tan et al., 2009; Poirier et al., 2011; Chandramathi et al., 2012; Jiménez-González et al., 2012; Mirjalali et al., 2015; Yersal et al., 2016). In China, no studies involve *E. bieneusi* infection in cancer patients and no molecular data are available on *Blastocystis* infection in this population. Thus, a cross-sectional molecular epidemiological study of *E. bieneusi* and *Blastocystis* infection in cancer patients was conducted to determine the prevalence, genotypes/subtypes of both pathogens in cancer patients by sequencing and analyzing the ITS region of the rRNA gene and the barcode region of the SSU rRNA gene, respectively. The relationships between *E. bieneusi* and *Blastocystis* infections and diarrhea were explored as well as the role that chemotherapy plays in the infection of both pathogens. Meanwhile, the potential of zoonotic transmission was assessed.

## Materials and methods

### Ethics statement

This research study was approved by the Medical Ethics Review Committee of Harbin Medical University. All the cancer patients undergoing chemotherapy gave their written informed consent for chemotherapy. Medical Ethics Review Committee of Harbin Medical University exempted the individual informed consent targeting molecular identification of *E. bieneusi* and *Blastocystis* in cancer patients based on the fact that only fecal specimens were analyzed for the two pathogens in the present study and the patients' personal information did not appear here.

### Specimen collection and DNA extraction

Fecal specimens of 381 cancer patients (one each) were collected from the Third Affiliated Hospital (namely Tumor Hospital) of Harbin Medical University, in China during April 2016 to January 2017. 188 patients were diagnosed newly (41 with diarrhea and 147 without diarrhea) and 193 patients undergoing chemotherapy (69 with diarrhea and 124 without diarrhea), including lung cancer (*n* = 90), stomach cancer (*n* = 88), colorectal cancer (*n* = 49), liver cancer (*n* = 47), esophagus cancer (*n* = 29), breast cancer (*n* = 28), and hematologic cancer (*n* = 22) as well as other types of cancer (*n* = 28). Of 381 patients, 220 were males and 161 were females with their ages ranging from 25 to 84 years; meanwhile, 237 patients were from urban areas while 144 from rural areas (Table 1).

**Table 1:** Prevalence of *E. bieneusi* and *Blastocystis* in cancer patients by age, gender, residence, clinical symptom, and cancer type.

**Group**		**Examined no**.	***E. bieneusi***		***Blastocystis***	
			**Positive no. (%)**	**χ^2^/*p*-value**	**Positive no. (%)**	**χ^2^/*p*-value**
Age (years)	24–44	42	0	−/0.32^a^	2 (4.8)	0.39/0.82
	45–65	269	3 (1.1)		20 (7.4)	
	66–86	70	2 (2.9)		5 (7.1)	
Gender	Male	220	2 (0.9)	0.12/0.72	12 (5.5)	2.11/0.15
	Female	161	3 (1.9)		15 (9.3)	
Residence	Urban	237	3 (1.3)	0.13/0.72	16 (6.8)	0.11/0.74
	Rural	144	2 (1.4)		11 (7.6)	
Symptom	Diarrhea	110	4 (3.6)	4.17/0.04	14 (12.7)	7.47/0.01
	Non-diarrhea	271	1 (0.4)		13 (4.8)	
Cancer type	Lung	90	2 (2.2)		8 (8.9)	3.02/0.88
	Stomach	88	1 (1.1)		7 (8.0)	
	Colorectal	49	1 (2.0)		4 (8.1)	
	Liver	47	1 (2.1)		3 (6.4)	
	Esophagus	29	0		2 (6.9)	
	Breast	28	0		2 (7.1)	
	Haematologic	22	0		1 (4.5)	
	Others	28	0		0	

a *Fisher's exact test*.

Genomic DNA was extracted directly from 180 to 200 mg fecal specimen using a QIAamp DNA Stool Mini Kit (QIAgen, Hilden, Germany) according to the manufacturer-recommended procedures. To obtain high yield of DNA, the lysis temperature was increased to 95°C according to the manufacturer's suggestion. DNA was finally eluted stored at −20°C in a freezer until further use in PCR analysis.

### PCR amplification

Each of the DNA specimens was analyzed for the presence of *E. bieneusi* by nested PCR amplifying an ~390 bp fragment of the rRNA gene including 243 bp ITS region. The primer sequences and the cycling parameters in nested PCR analysis were used as previously described (Buckholt et al., 2002). Meanwhile, a ~600 bp fragment (barcode region) of the SSU rRNA gene of *Blastocystis* was amplified. The primer sequences and the cycling parameters in PCR analysis were used as previously described (Scicluna et al., 2006).

TaKaRa Taq DNA Polymerase (TaKaRa Bio Inc., Tokyo, Japan) was used in all the PCR amplifications. All the PCR amplifications were run with non-template water control. All the DNA specimens were analyzed twice using 2 μL by PCR. All the PCR products were separated in 1.5% agarose gel electrophoresis and visualized under UV light after ethidium bromide staining.

### Nucleotide sequencing and molecular analysis

All the PCR products of the expected size were directly sequenced with their respective primers after being purified on an ABI PRISM 3730 XL DNA Analyzer by Sinogeno-max Biotechnology Co., Ltd. (Beijing, China), using the Big Dye Terminator v3.1 Cycle Sequencing Kit (Applied Biosystems, USA). Sequence accuracy was confirmed by two-directional sequencing and by sequencing at least two new PCR products for some DNA preparations from which we obtained sequences with nucleotide substitutions, deletions or insertions.

Nucleotide sequences obtained in the present study were subjected to BLAST searches (http://www.ncbi.nlm.nih.gov/blast/) and then aligned and analyzed with each other and reference sequences downloaded from GenBank database using the program Clustal X 1.83 (http://www.clustal.org/) to determine genotypes of *E. bieneusi* isolates and subtypes of *Blastocystis* isolates. The genotypes of *E. bieneusi* obtained in this study were given the first published name if they were identical to known genotypes in GenBank database (Santín and Fayer, 2009). If not, the genotypes were considered novel genotypes. In this study, all the novel and known genotypes were identified only based on 243 bp of the ITS region of the rRNA gene of *E. bieneusi* according to the established nomenclature system (Santín and Fayer, 2009). Subtypes of *Blastocystis* isolates were identified by determining the exact match or the closest similarity according to terminology for *Blastocystis* subtypes-a consensus (Stensvold et al., 2007).

Novel nucleotide sequences obtained in the present study were deposited in the GenBank database.

### Phylogenetic and statistical analyses

All the aligned nucleotide sequences of the ITS region of *E. bieneusi* were implemented in the software Mega 5 (http://www.megasoftware.net/). Phylogenetic relationship of novel genotypes obtained in the present study to known ones downloaded in GeneBank databases was explored by constructing a neighbor-joining tree based on the evolutionary distances calculated by the Kimura 2-parameter model. The genogroup of novel ITS genotype of *E. bieneusi* was designated. The reliability of the trees was assessed using the bootstrap analysis with 1,000 replicates.

Fisher's exact test and Pearson chi-square (χ^2^) tests based on the Statistical Package for the Social Sciences (SPSS) 19.0 were used to determine statistical significance in the present study.

## Results

### Infection rates of *E. bieneusi* and *Blastocystis* by demographic characteristics

In total, 381 stool specimens were collected from cancer patients (220 male, 161 female) with average age of 56 (ranging in age from 25 to 84 years) (Table 1).

Infection rates for *E. bieneusi* and *Blastocystis* differed in the groups divided by age, gender and residence. However, all the differences in infection rates had no statistical significance. *E. bieneusi* was only found in the age groups of 45–65 years (1.1%, 3/269) and 66–86 years (2.9%, 2/70). *Blastocystis* could be seen in all the three age groups, with the peak in 45–65 years (7.4%, 20/269). Females had a higher infection rate than males either for *E. bieneusi* (1.9 and 0.9%) or for *Blastocystis* (9.3 and 5.5%). *E. bieneusi* and *Blastocystis* were observed more commonly in patients from rural regions (1.4 and 7.6%) than those from urban regions (1.3 and 6.8%). Meanwhile, *E. bieneusi* was found in cancer patients: lung (2.2%, 2/90), stomach (1.1%, 1/88), colorectal (2.0%, 1/49), and liver (2.1%, 1/47); and *Blastocystis* was detected in cancer patients: lung (8.9%, 8/90), stomach (8.0%, 7/88), colorectal (8.1%, 4/49), liver (6.4%, 3/47), esophagus (6.9%, 2/29), breast (7.1%, 2/28) and hematologic (4.5%, 1/22). No relationship was observed between *Blastocystis* and types of cancer, and it was impossible to evaluate the relationship between *E. bieneusi* and types of cancer due to only five positive cases (Table 1).

In general, *Blastocystis* was more prevalent than *E. bieneusi*, accounting for five and 27 specimens PCR-positive for *E. bieneusi* (1.3%, 5/381) and for *Blastocystis* (7.1%, 27/381), respectively. Co-infection of *E. bieneusi* and *Blastocystis* was observed in a cancer patient. *E. bieneusi* was found in 2.1% of 193 patients undergoing chemotherapy and 0.5% in 188 patients newly diagnosed. *Blastocystis* was detected in 9.8% (19/193) and 4.3% (8/188) in the two groups, respectively (Table 2).

**Table 2:** Prevalence and distribution of *E. bieneusi* genotypes and *Blastocystis* subtypes in cancer patients by treatment.

**Groups (Examined no.)**	***E. bieneusi***			***Blastocystis***		
	**Positive no. (%)**	**χ^2^/*p*-value**	**Genotype/s (*n*)**	**Positive no. (%)**	**χ^2^/*p*-value**	**Subtype/s (*n*)**
**CHEMOTHERAPY**						
Diarrhea (69)	3 (4.3)	1.27/0.26	Type D (2), Novel (1)	11 (15.9)	4.5/0.03	ST1 (6), ST3 (5)
Non-diarrhea (124)	1 (0.8)		Type D (1)	8 (6.5)		ST1 (3), ST3 (5)
Subtotal (193)	4 (2.1)		Type D (3), Novel (1)	19 (9.8)		ST1 (9), ST3 (10)
**NON-CHEMOTHERAPY**						
Diarrhea (41)	1 (2.4)	–/0.22^a^	Type D (1)	3 (7.3)	0.44/0.51	ST1 (2), ST3 (1)
Non-diarrhea (147)	0			5 (3.4)		ST1 (1), ST3 (4)
Subtotal (188)	1 (0.5)		Type D (1)	8 (4.3)		ST1 (3), ST3 (5)
Total (%)	5 (1.3)		Type D (4), Novel (1)	27 (7.1)		ST1 (12), ST3 (15)

a *Fisher's exact test*.

### Relationships between *E. bieneusi* and *Blastocystis* infections and diarrhea

The cancer patients with diarrhea had significantly higher infection rates than those without diarrhea either for *E. bieneusi* (3.6 vs. 0.4) or for *Blastocystis* (12.7% vs. 4.8%) (Table 1). In chemotherapy group, the infection rates of *E. bieneusi* and *Blastocystis* were higher in the patients with diarrhea (4.3 and 15.9%) than those without diarrhea (0.8 and 6.5%), and significant relationship of *Blastocystis* to diarrhea was observed here (χ^2^ = 4.5, *p* < 0.05) (Table 2). No statistical difference was found between *E. bieneusi* and *Blastocystis* infections and diarrhea in the patients without treatment (*p* > 0.05).

### *E. bieneusi* genotyping and phylogenetic analysis

**Figure 1:**
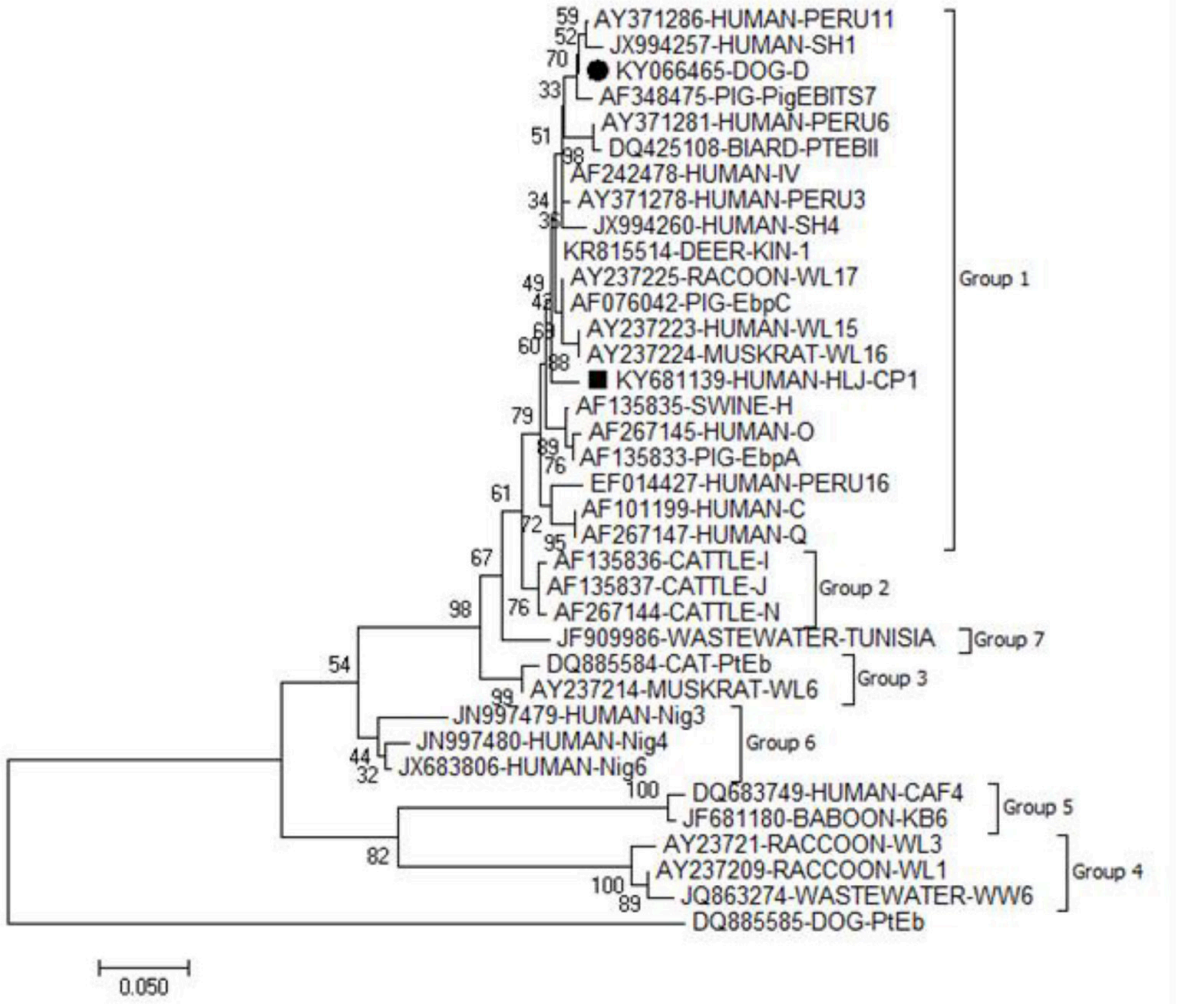
Phylogenetic relationship of the genotypes of *E. bieneusi*. The relationship between the genotypes of *E. bieneusi* identified in the present study and known genotypes of *E. bieneusi* deposited in the GenBank was inferred by a neighbor-joining analysis of ITS gene sequences based on genetic distance by the Kimura 2-parameter model. Each sequence is identified by its accession number, host origin, and genotypes. The numbers on the branches are percent bootstrapping values from 1,000 replicates. The circle and square filled in black indicate known and novel genotypes identified in the present study, respectively.

All the five *E. bieneusi* isolates were successfully genotyped by sequence analysis of the ITS region of the rRNA gene. Four ITS gene sequences were identical to one another and had 100% homology with that of genotype D (KU847361). The remaining one was not described previously, and named as HLJ-CP1 (KY681139), which had 98.35% homology with the genotype KIN-1 (DQ683746). In a phylogenetic analysis, genotypes D and HLJ-CP1 obtained here belonged to zoonotic group 1 (Figure 1).

### *Blastocystis* subtyping

By sequence analysis of the barcode region of the SSU rRNA gene of 27 *Blastocysti*s isolates, two different *Blastocystis* subtypes were identified: ST1 (*n* = 12) and ST3 (*n* = 15). ST1 (66.7%, 8/12) was observed to be more prevalent than ST3 (40.0%, 6/15) in patients with diarrhea (Table 2).

Five representative ST1 sequences were obtained out of 12 *Blastocysti*s isolates, with 11 polymorphic sites being observed. Among them, two ST1 sequences had 100% similarity with the reference sequences AB107962 and EU445492, respectively. The remaining three ST1 sequences were not described previously. Two sequences (KY681140 and KY681141) had 99.32% similarity with the reference sequences AB070993 and EU445488, respectively, while the remaining one (KY681142) had 99.66% similarity with the reference sequence EU445490 (Table 3).

**Table 3:** Nucleotide variations in the barcode region of the SSU rRNA gene of *Blastocystis* ST1 isolates in this study.

**Accession no. (Isolate no.)**		**Nucleotide at position (Barcode region)^b^**										
		**74**	**75**	**76**	**77**	**78**	**130**	**177**	**266**	**477**	**480**	**481**
Known^a^	AB107962 (4)	T	G	T	T	A	G	A	A	T	A	C
	EU445492 (4)	A	T	C	A	A	G	A	A	T	C	A
Novel	KY681140 (1)	G	T	A	G	T	A	G	A	T	C	A
	KY681141 (2)	G	T	A	G	T	A	A	G	G	A	C
	KY681142 (1)	T	G	T	T	A	G	A	A	T	C	A

a *Accession numbers indicating these known sequences downloaded from GenBank which have 100% similarity with those obtained in the present study*.

b *Nucleotide position numbers according to AB107962, with the beginning of the SSU rRNA gene as position no. 1*.

Among 15 *Blastocysti*s isolates, 13 and two ST3 sequences were identical to the reference sequences KT374026 and JF792494, respectively, with only one base variation being seen between the two representative ST3 sequences.

## Discussion

*E. bieneusi* and *Blastocystis* as two common opportunistic protozoa have been found in humans, especially in immunocompromised individuals, such as AIDS patients, organ transplant recipients and cancer patients. The present study is the first report of the prevalence and characterization of *E. bieneusi* and *Blastocystis* by molecular techniques in cancer patients in China.

In the present study, overall infection rates of *E. bieneusi* and *Blastocystis* were 1.3% (5/381) and 7.1% (27/381), respectively. Both of them were lower than those in previous reports from cancer patients, with *E. bieneusi* in Mexico (40%, 4/10), Turkey 23.7% (22/93), and Iran (1.7%, 4/234), and *Blastocystis* in France (16%, 15/94), Malaysia (21.1%, 43/204), and Turkey (10.8%, 25/232) (Poirier et al., 2011; Jiménez-González et al., 2012; Kumarasamy et al., 2014; Hamamc1 et al., 2015; Mirjalali et al., 2015; Yersal et al., 2016). In fact, infection rates of these two pathogens vary from country to country and within different communities of the same country. Infection rates are related to many factors. In fact, infection rates of these two pathogens vary from country to country and within different communities of the same country. Infection rates are related to many factors. Except for influence of immune status of hosts, main reasons might be related to local inhabitants' dietary habits and hygiene conditions. In the present study, the lower infection rates might be related to that local inhabitants have a habit of drinking boiled water and eating cooked food/vegetables. *E. bieneusi* was only found in the age groups of 45–65 years and 66–86 years, while *Blastocystis* could be seen in all the three age groups, with the peak prevalence in 45–65 years. Higher infection rates of *E. bieneusi* and *Blastocystis* were observed among females and patients from rural areas. There was no statistical significance between the groups based on age, gender and residence. However, a recent study of *Blastocystis* subtypes in cancer patients indicated that infection rates were significantly higher among males and patients from urban areas (Yersal et al., 2016).

Diarrhea is a common clinical symptom caused by *E. bieneusi*, especially in immunocompromised individuals. Some scholars found that there was significant relationship between *E. bieneusi* infection and diarrhea in HIV-positive individuals (Ojuromi et al., 2012; Agholi et al., 2013a; Khanduja et al., 2017). A similar finding was seen in cancer patients in the present study. However, no significant difference in occurrence rate was found in chemotherapy group. It might be related to the less number of the positive cases obtained in the present study. In fact, chemotherapy drugs, which are known to be cytotoxic, are considered to possibly down-regulate patient immune system and may trigger latent intestinal parasitic infections (Solomayer et al., 2003; Koivusalo and Hietanen, 2004).

*Blastocystis* sp. can be found in both symptomatic and asymptomatic patients. However, there is increasing evidence to suggest that immunocompromised individuals are more prone to suffer from *Blastocystis*-related diarrheal illness (Prasad et al., 2000; Taşova et al., 2000). In the present study, *Blastocystis* was detected more commonly in cancer patients with diarrhea. The same result was observed in the patients undergoing chemotherapy. In the present study, due to the patient lifestyle, diet intake and home dwellings unchanged throughout the treatment period, it was supposed the patients might have the *Blastocystis* infection before treatment, and diarrhea did not begin to occur until they had undergone chemotherapy. Chemotherapy in cancer patients is reported to possibly cause changes in intestinal microbiota (Touchefeu et al., 2014). Additionally, *Blastocystis* is considered to be related to particular bacterial communities and individuals with certain intestinal flora are susceptible to *Blastocysti*s infection (Andersen et al., 2015). However, Yersal et al. drew an opposite conclusion that *Blastocystis* was found more frequently in cancer patients without diarrhea (Yersal et al., 2016). Definitive conclusion needs to be confirmed by more epidemiological data of *Blastocystis* infection in humans in the future.

In the present study, there was no relationship observed between *Blastocystis* and types of cancer. However, *Blastocystis* was observed to have a higher frequency in patients with lung cancer than the other cancer types (Yersal et al., 2016). So far, no reports have been available on the relationship between *E. bieneusi* and types of cancer, which was related to few positive cases for *E. bieneusi*, including the present data.

In the present study, two *E. bieneusi* genotypes were identified, including a known genotype D (*n* = 4) and a novel genotype (HLJ-CP1). In a phylogenetic analysis, genotype HLJ-CP1 was clustered into zoonotic group 1, suggesting the possibility of zoonotic transmission. Genotype D (syn. CEbc, PigEBITS9, WL8, Peru9, and PtEb VI) is more common in humans than other genotypes, and has a wide geographical distribution (Matos et al., 2012). This genotype has also been found in transplant recipients (Galván et al., 2011; Pomares et al., 2012; Agholi et al., 2013b; Mirjalali et al., 2015; Kicia et al., 2016) and cancer patients (Mirjalali et al., 2015), and showed predominance in the patients infected with HIV (Sulaiman et al., 2003; Leelayoova et al., 2006; Sokolova et al., 2011). In China, genotype D has been detected in children (Wang et al., 2013a) and HIV-positive patients and HIV-negative individuals (Wang et al., 2013b). It is known that genotype D also has a wide host ranges (Yang et al., 2016). Besides humans, it has been detected in at least 15 animal species (Mori et al., 2013; Zhao et al., 2015). In the investigated areas, genotype D has been isolated from pigs, sheep, goats, cattle, foxes, raccoon dogs and rabbits and is dominant in pigs and foxes (Yang et al., 2016). Pigs are the most important economic animals and appear in a great number in the investigated areas. The large output of feces and unscientific practices can result in environmental contamination. People can acquire *E. bieneusi* infection by ingesting infective spores in vegetables and water contaminated from pig manure. Previous studies also showed contact with pigs was a risk factor for acquisition of *E. bieneusi* infection (Leelayoova et al., 2009). Based on the report of high prevalence of *E. bieneusi* and large percentage of zoonotic genotypes including genotype D in pigs in the investigated area (Zhao et al., 2014b), it was speculated that cancer patients might acquire *E. bieneusi* infection from pig manure sources.

In the present study, 12 and 15 *Blastocystis* isolates were identified as subtypes ST1 and ST3, respectively. Molecular epidemiological data have showed that nine STs have been found in humans worldwide. ST3 is the most common subtype in humans and shows a wide geographical distribution (Stensvold, 2013). Recent three studies of *Blastocystis* infection in cancer patients also indicated that ST3 was more common than ST1 (Tan et al., 2009; Kumarasamy et al., 2014; Yersal et al., 2016). However, a survey conducted in France revealed ST4 was the most prevalent subtype in cancer patients, followed by ST3 and ST7 (Poirier et al., 2011).

Epidemiological data have demonstrated the presence of ST1 and ST3 in pigs, cattle, dogs and cats (Clark et al., 2013). Furthermore, ST1 is also found in monkeys, apes, and baboons (Parkar et al., 2007) while ST3 in rats (Ramírez et al., 2014). Thus, the animals above may act as potential reservoir hosts and contributes to environmental pollution and continuous transmission of this disease. In fact, in the present study, ST1 and ST3 sequences of the barcode region of *Blastocystis* were also found in animal hosts in Japan: a pig (AB107961) and an ape (AB107967) for ST1; cattle (AB107965) and a pig (AB107963) for ST3. Due to few data in China and no data available in the investigated areas on subtyping of animal-derived *Blastocystis* isolates, it is unclear on true pollution/contamination sources and transmission routes of human *Blastocystis* infection.

In the present study, ST1 (66.7%, 8/12) was more prevalent than ST3 (40.0%, 6/15) in cancer patients with diarrhea. Currently, one of the key questions on pathogenicity of *Blastocystis* is whether disease is STs related. Although a few studies have been carried out; however, no consensus is reached on this issue. Five STs (ST1, ST2, ST3, ST4, and ST6) have been found in humans with clinical symptoms (Tan, 2008). Some studies supported that ST1 is associated with disease (Kaneda et al., 2001; Yoshikawa et al., 2004; Yan et al., 2006). In contrast, ST3 is reported to be predominant in symptomatic patients (Dogruman-Al et al., 2008; Tan et al., 2008; Moosavi et al., 2012). In addition, intra subtype variations in pathogenicity have also been noted, that is, not all the strains of a particular subtype are pathogenic (Scanlan, 2012). Due to the less number of *Blastocysti*s isolates were analyzed in our study, we could not draw a conclusion about the relationship between intra-subtype variations and virulence.

In conclusion, the present study is the first report of occurrence and genotyping of *E. bieneusi* and subtyping of *Blastocystis* in cancer patients in China. The finding of higher infection rates of the two parasites in cancer patients undergoing chemotherapy suggests the necessarity to screen intestinal microorganisms causing diarrhea in this population. The *E. bieneusi* genotypes and *Blastocystis* subtypes described in this study with novel sequences for both parasites in the cancer patients, as well as, their presence in animal hosts (Stensvold, 2013; Yang et al., 2016) would support the potential of zoonotic transmission. Risk factors will be assessed in the future by carrying out molecular epidemiological surveys of *E. bieneusi* and *Blastocystis* in wild and domestic animals and environmental samples.

## Author contributions

AL, JC, and WZhang designed this study. GR, WZhao, and ZY performed the experiments. GR and WZhao analyzed the data. YuS and YiS Contributed reagents/materials. WZhang, AL, and GR wrote the manuscript and prepared the tables and figures. All authors edited the manuscript.

### Conflict of interest statement

The authors declare that the research was conducted in the absence of any commercial or financial relationships that could be construed as a potential conflict of interest.
